# Emerging Clinical Complications of Alpha-Gal Syndrome: A Scoping Review

**DOI:** 10.7759/cureus.113589

**Published:** 2026-07-29

**Authors:** Vishnu S Vundamati, Shaan R Patel, Riyana Patel, Nisa Eriskin, Brielle Tila-Cohen, Caiden J Friedrich, Advait Gupta, Raghavee Neupane, Sebastian Arango, Marc M Kesselman

**Affiliations:** 1 Biological Sciences, Nova Southeastern University, Davie, USA; 2 Medicine, Nova Southeastern University Dr. Kiran C. Patel College of Osteopathic Medicine, Davie, USA; 3 Public Health, Nova Southeastern University, Davie, USA; 4 Kinesiology, Rutgers University, New Brunswick, USA; 5 Allergy and Immunology, Nova Southeastern University Dr. Kiran C. Patel College of Osteopathic Medicine, Davie, USA; 6 Rheumatology, Nova Southeastern University Dr. Kiran C. Patel College of Osteopathic Medicine, Davie, USA

**Keywords:** allergy, alpha-gal syndrome, lone star tick, mammalian meat allergy, red meat allergy

## Abstract

Alpha-gal syndrome (AGS) is characterized by an IgE-mediated immune response against galactose-α-1,3-galactose (α-gal). In the United States, the principal vector is the lone star tick, whose range has expanded nationwide. A lone star tick bite delivers α-gal into human skin via its saliva, triggering an IgE response in susceptible individuals. Vomiting, urticaria, and anaphylaxis may occur immediately or hours after exposure. However, perioperative ingredients, such as gelatin, monoclonal antibodies, stearic acid, and other mammalian-derived products, have triggered anaphylactic shock in patients with AGS. These nuances complicate AGS management as many cases go unrecognized. This review aims to look beyond traditional food-related reactions, characterize the range of presentations, and improve management of this increasingly recognized condition. The inclusion criteria required original research in English peer-reviewed journals between January 1, 2000, and March 22, 2026. Eligible studies included quantitative, qualitative, and mixed-method designs examining AGS acquisition. Relevant tick studies were included. Systematic reviews, scoping reviews, meta-analyses, case reports, series, and studies involving pregnant individuals or children, lacking full-text, duplicate publications, or overlapping datasets were excluded. Authors screened publications using Rayyan (Rayyan Systems Inc., Cambridge, USA). Discrepancies were resolved through discussion. Of the 729 records identified, 14 studies met the inclusion criteria. Understanding the diverse spectrum of allergic manifestations of this condition is vital. This review consolidates findings on AGS-related complications, including immediate and delayed anaphylaxis, hypersensitivity reactions to mammalian-derived medical products, and blood transfusions. However, the available evidence is primarily derived from observational studies conducted in the United States, which may limit the generalizability of current findings. By highlighting the broader implications of AGS, this review underscores awareness in acute and perioperative settings.

## Introduction and background

**Figure 1:**
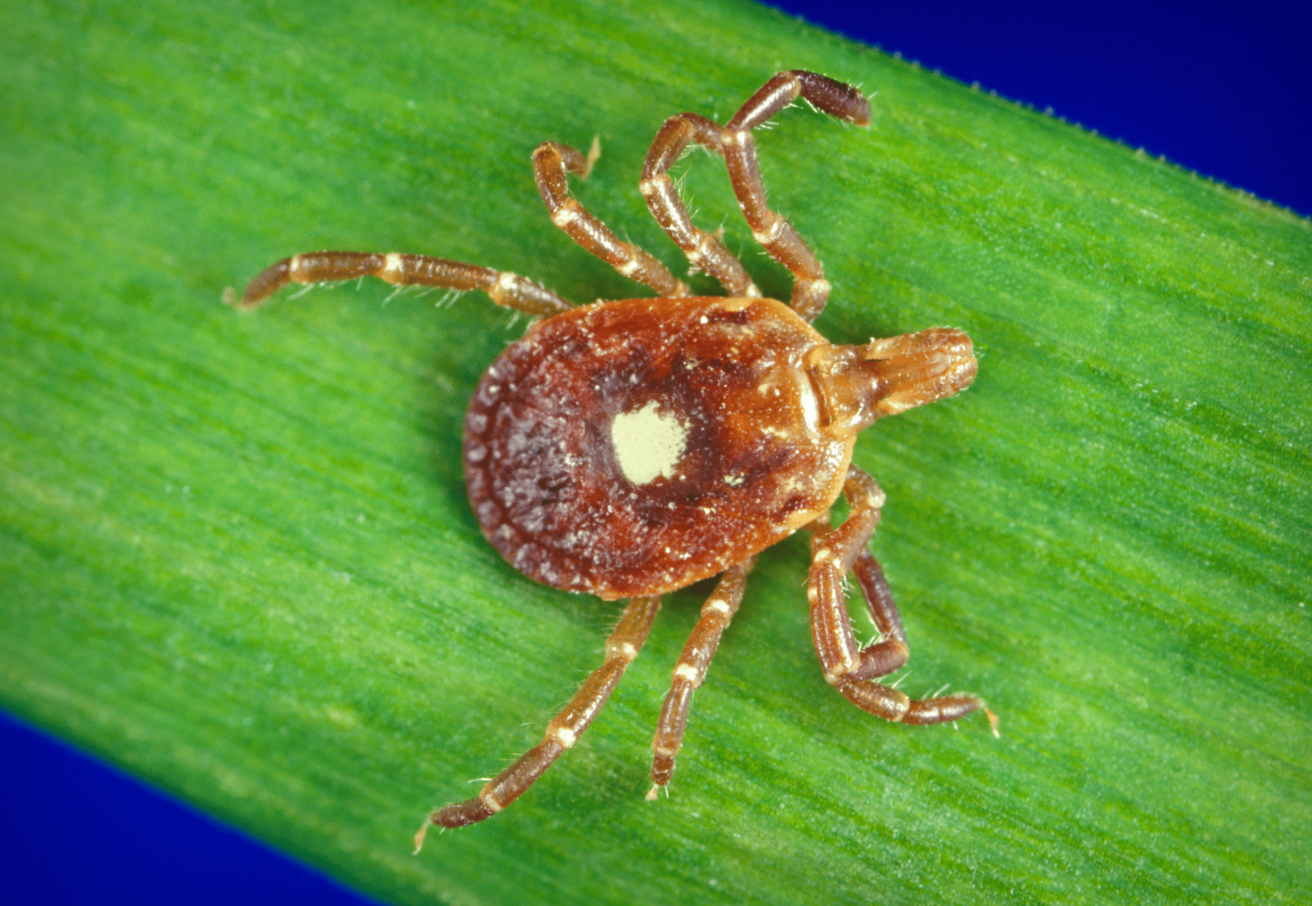
Lone star tick (Amblyomma americanum), primary vector of AGS in the United States. Image courtesy of the Centers for Disease Control and Prevention (CDC) Public Health Image Library (PHIL #4407), public domain, via Wikimedia Commons [2]. AGS: alpha-gal syndrome

Alpha-gal syndrome (AGS) is an emerging allergic condition characterized by an immunoglobulin E (IgE)-mediated hypersensitivity to galactose‑alpha‑1,3‑galactose (α‑gal), a carbohydrate found in mammalian meat. Unlike traditional food allergies, AGS is uniquely associated with sensitization following bites from the lone star tick (*Amblyomma americanum*), shown in Figure 1, which triggers the development of α‑gal-specific IgE antibodies. As a result, affected individuals may experience allergic reactions after consuming red meat or exposure to mammalian‑derived products. Notably, AGS differs from conventional food allergies in that symptoms are often delayed, occurring several hours after exposure, and may include urticaria, gastrointestinal symptoms, or even life‑threatening anaphylaxis [1].

The prevalence of AGS has increased significantly in the United States in recent years, largely correlating with the geographic expansion of the lone star tick. Large‑scale epidemiological data identified tens of thousands of suspected cases nationwide, with a substantial rise in α‑gal IgE testing concentrated in Southeastern and lower Midwestern states [3]. This is reflective of the expanding geographic range of the lone star tick. Further studies show that α‑gal sensitization varies regionally, with a higher prevalence in areas with the highest tick exposure [4]. 

Dietary exposure to α‑gal is a common form of sensitization; however, exposure to certain medications, biologics, and blood components has also been associated with allergic responses in sensitized individuals. Although these manifestations have been reported in case studies and individual studies, evidence remains fragmented, with a limited synthesis of unconventional exposures associated with AGS. This expanding clinical spectrum underscores the complexity of AGS and the need to improve characterization of its manifestations. This review aims to look beyond traditional food-related reactions, characterize the range of presentations, and improve management of this increasingly recognized condition.

## Review

Methods

Search Strategy

A comprehensive search was conducted using the following electronic databases: PubMed, Google Scholar, and Embase. The search was conducted using a predefined list of controlled vocabulary terms and keywords. Search terms included “galactose alpha-1,3-galactose,” “alpha-gal,” “alpha-gal syndrome,” “*Amblyomma americanum*,” “Lone Star tick,” “sensitization," “IgE,” “anaphylaxis,” “delayed anaphylaxis,” “red meat allergy,” “urticaria,” “heparin-,” “gelatin-,” and “bovine-derived.” These descriptors were applied using the standard Boolean operators (AND, OR, NOT) in accordance with database search engine mechanics to capture as many relevant articles as possible. The complete database-specific search strategies, including search dates, are provided in the Appendices.

Inclusion and Exclusion Criteria

Initial data screening was performed independently by seven authors (V.V., S.P., R.P., N.E., B.C., C.F., and A.G.), and articles that met the inclusion criteria were included in the preliminary review. Inclusion criteria were (1) studies assessing reactions to bovine-, porcine-, or heparin-derived medical products in patients with AGS, (2) AGS-specific IgE in relation to anaphylaxis, delayed anaphylaxis, transfusion reactions, and urticaria, and (3) observational, interventional, or experimental human studies: cohort, case-control, cross-sectional, retrospective, prospective, randomized controlled trials, and studies using human-derived specimens. Exclusion criteria were (1) studies focused on species besides *A. americanum*, (2) studies evaluating idiopathic anaphylaxis reactions without suspected AGS association, and (3) non-English publications, review articles, systematic reviews, meta-analyses, conference abstracts, gray literature, editorials, commentaries, and animal studies.

Data Extraction and Reporting

**Figure 2:**
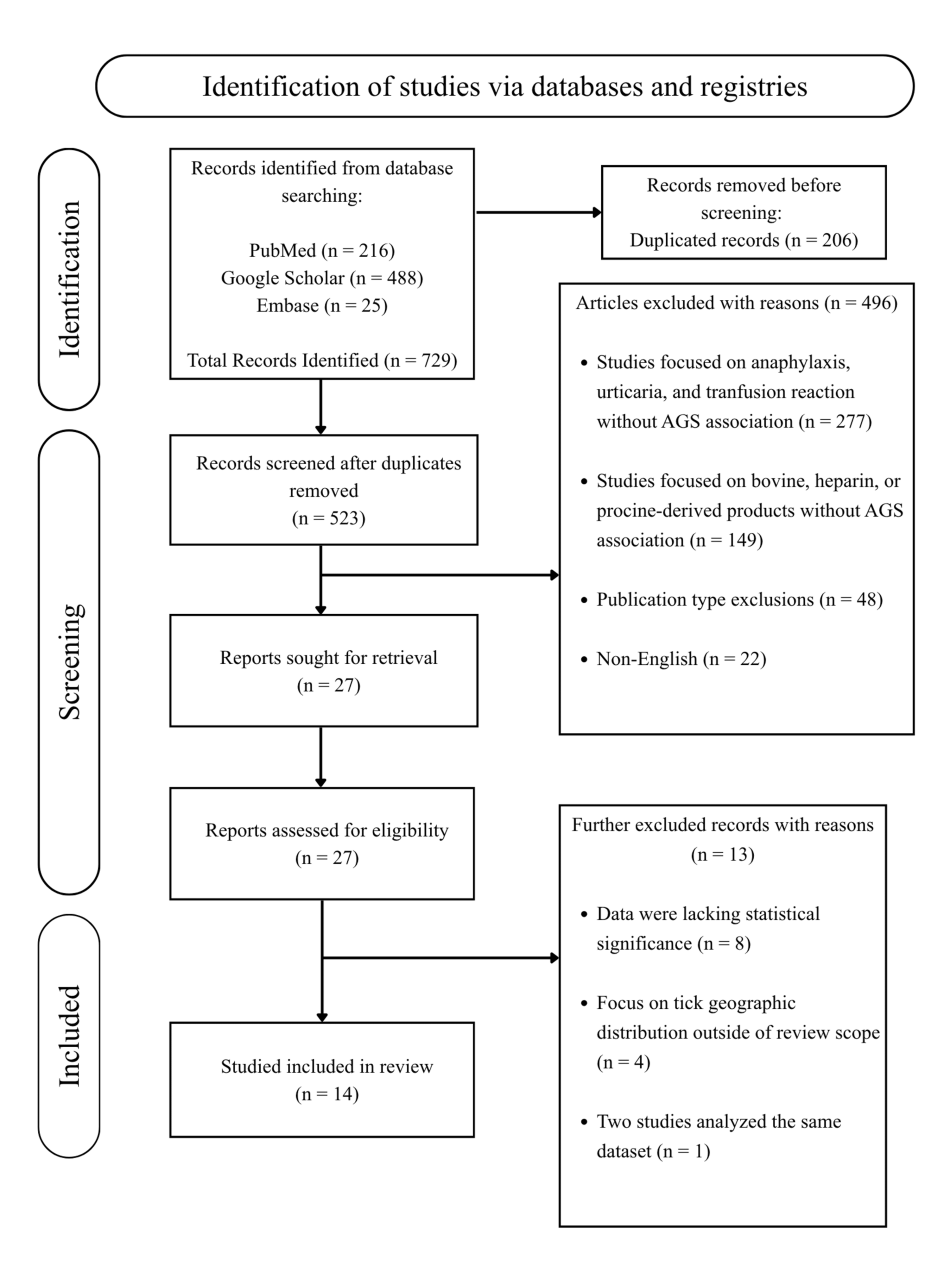
PRISMA diagram Flow diagram created by the authors reflecting the study's selection process in accordance with the PRISMA-ScR guidelines [6]. PRISMA-ScR: Preferred Reporting Items for Systematic reviews and Meta-Analyses extension for Scoping Reviews

A total of 729 articles were identified in the initial screening. Utilizing the Rayyan software (Rayyan Systems Inc., Cambridge, USA), 206 duplicates were detected [5]. After eliminating duplicate articles, the authors were left with 523 articles. After the initial data screening, the seven authors (V.V., S.P., R.P., N.E., B.C., C.F., and A.G.) independently performed a secondary screen of 27 articles to determine suitability. Any discrepancies regarding the inclusion of articles were resolved by discussion to achieve a consensus of 14 articles for the review. The review utilized the PRISMA Extension for Scoping Reviews (PRISMA-ScR) guidelines, as shown in Figure 2. Consistent with scoping review methodology, a formal risk-of-bias assessment was not performed, as the objective of this review was to characterize reported clinical manifestations rather than to evaluate intervention effectiveness or to determine the methodological quality of individual studies.

Results

Tripathi et al. [7] described patients initially diagnosed with idiopathic anaphylaxis who were later found to have delayed anaphylaxis associated with red meat consumption. Nwamara et al. [8] found that heparin products were tolerated in patients with documented AGS, but unfractionated heparin resulted in more reactions. Hawkins et al. [9] demonstrated that patients who reacted to intravenous heparin had significantly higher preoperative IgE titers than their counterparts, establishing titer levels as a potential risk evaluator. Commins et al. [10] reported associations between tick exposure and α-gal sensitization. Platts-Mills et al. [11] and Commins et al. [12] focused on α-gal-specific IgE-mediated anaphylaxis outcomes. They jointly concluded that α-gal-specific IgE drives fatal anaphylaxis through a carbohydrate-directed mechanism, atypical among known allergens. Villar et al. [13] used a proteomic analysis to identify α-gal-modified protein content of secreted saliva in *A. americanum*. The identified proteins were screened against serum from patients with confirmed AGS to propose candidate disease biomarkers. Two studies addressed hypersensitivity reactions related to transfusion products, identifying α-gal-associated risks in plasma and platelet exposures [14,15]. Dunbar et al. [14] introduced the term "transfusion-related AGS (TRAGS)" to describe the risk posed to group O patients receiving group B plasma, as they noted similarities between the α-gal oligosaccharide and group B blood antigen. Gilstad et al. [15] described an anaphylactic outbreak to group B platelets in group O recipients, establishing α-gal sensitization as a cause of lethal transfusion reactions. Schmidle et al. [16] used basophil activation testing to show gelatin-containing vaccines for varicella, zoster, MMR, and rubella-elicited adverse responses in patients with AGS but not in patients with AGS tested with a non-gelatin formulation. Two studies addressed clinical management with an emphasis on dietary restrictions and quality-of-life implications in patients with AGS [17,18]. Kennedy et al. [18] emphasized awareness of the expanding range of *A. americanum*. Filip et al. [19] extended this concern to crotaline antivenoms, finding elevated adverse reactions to mammalian-derived biologics (CroFab and ANAVIP) in α-gal endemic regions. Mitchell et al. [20] reported associations between tick exposure and α-gal sensitization. Four studies focused on hypersensitivity to heparin, gelatin-containing vaccines, and other mammalian-derived biologics, highlighting unique allergic responses in sensitized individuals [7,8,16,19]. Three studies reported delayed α-gal-specific IgE-mediated anaphylaxis following mammalian meat ingestion or sensitization events [7,11,12]. Five articles addressed IgE sensitization and tick exposure-driven immune activation [10-13,20]. A summary of the articles used for this review is provided in Table 1.

**Table 1:** Summary of the articles used for this review

Reference	Study design	Outcome of interest	Main findings
Tripathi et al., 2014 [7]	Prospective observational clinical study	IgE-mediated anaphylaxis	Delayed anaphylaxis caused by IgE antibodies to galactose-α-1,3-galactose. Sensitization detected best with specific IgE testing.
Nwamara et al., 2022 [8]	Retrospective case-series	Hypersensitivity to mammalian-derived medical products	Heparin products tolerated by patients with α-gal allergies. One reaction to unfractionated heparin in a patient with high baseline α-gal IgE titers (24.20 kU/L).
Hawkins et al., 2021 [9]	Retrospective cohort study	Hypersensitivity to mammalian-derived medical products	Intravenous heparin triggered severe reactions. Associated with significantly higher baseline α-gal IgE titers (median 75 kU/L vs. 8 kU/L in nonreactors).
Commins et al., 2011 [10]	Prospective observational cohort study	IgE sensitization and tick exposure-driven immune activation	20-fold or greater increase in α-gal-specific IgE levels following *A. americanum* bites. Primary cause being ectoparasite exposure.
Platts-Mills et al., 2015 [11]	Observational clinical study (cross-sectional)	IgE sensitization, tick exposure-driven immune activation, and IgE-mediated anaphylaxis	Elevated α-gal-specific IgE levels, with allergic reactions occurring three to six hours after ingesting red meat. Link between α-gal-specific IgE antibodies and delayed anaphylaxis.
Commins et al., 2016 [12]	Prospective observational cohort study	IgE sensitization, tick exposure-driven immune activation, and IgE-mediated anaphylaxis	Elevated α-gal IgE antibodies, with anaphylactic reactions occurring three to six hours after red meat ingestion. Strong correlation between IgE presence and delayed allergic responses to mammalian meat.
Villar et al., 2021 [13]	Experimental laboratory study (proteomic analysis using serum of patients with AGS)	IgE sensitization and tick exposure-driven immune activation	Identified α-gal epitopes on multiple N-linked glycoproteins in tick salivary glands and saliva.
Dunbar et al., 2025 [14]	Retrospective cohort study	Hypersensitivity to plasma/platelets/transfusion	ABO-mismatched plasma and platelet transfusions can increase sensitization to α-gal. Potential mechanism for transfusion-related allergic reactions in sensitized patients.
Gilstad et al., 2023 [15]	Retrospective observational cohort study (investigation of outbreak of transfusion reactions)	Hypersensitivity to plasma/platelets/transfusion	Anaphylactic transfusion reactions linked to group B blood products and mammalian-derived antigens present in plasma and platelets. Correlated with elevated α-gal IgE levels.
Schmidle et al., 2021 [16]	Experimental case-control study (basophil activation testing in patients with AGS)	Hypersensitivity to mammalian-derived medical products	Strong basophil activation when exposed to gelatin-containing vaccines, whereas the non-gelatin vaccines produced negative results.
Sempa et al., 2025 [17]	Observational cross-sectional study	Clinical management	Dietary restrictions and quality-of-life impact associated with AGS. Avoidance of mammalian-derived foods and medical products to prevent reactions.
Kennedy et al., 2021 [18]	Observational cross-sectional study	Clinical management	Expanding lone star tick populations highlight the need for proactive patient education on tick-bite prevention.
Filip et al., 2026 [19]	Comparative observational study	Hypersensitivity to mammalian-derived medical products	Anaphylactic reactions associated with antivenom administration in sensitized patients. Increased concern for antivenom reactions in α-gal endemic regions.
Mitchell et al., 2020 [20]	Prospective cohort study	IgE sensitization and tick exposure-driven immune activation	*Amblyomma americanum* bites induce α-gal-specific IgE. Mechanisms connecting tick bites with mammalian meat allergy.

Discussion

Tick-Mediated Sensitization

Tick exposure is the primary initiating event in the development of AGS and provides insight into the immunologic mechanism. Prospective studies demonstrated significant increases in α-gal-specific IgE levels following tick bites, strongly implicating *A. americanum* exposure as the initiating event [10]. Through mechanistic studies, such as the analysis of tick salivary glands and saliva, α-gal epitopes have been identified on multiple glycoproteins, supporting the hypothesis that tick-derived α-gal antigens stimulate IgE production in susceptible individuals [13]. Additionally, repeated tick bites may amplify α-gal-specific IgE sensitization, contributing to persistent immune activation and continued susceptibility to AGS manifestations [20].

Delayed Anaphylaxis

A severe manifestation of AGS is delayed-onset anaphylaxis following the ingestion of mammalian meat. This presentation differs from immediate allergic reactions. Patients in clinical investigations showed elevated α‑gal-specific IgE levels, with allergic reactions occurring approximately three to six hours after red meat consumption, confirming a strong correlation between IgE sensitization and delayed anaphylactic responses [11,12]. This delayed timing is a defining feature of AGS and may lead to misdiagnosis or classification as idiopathic anaphylaxis. One study demonstrated that many patients, initially diagnosed with unexplained allergic reactions, later tested positive for α‑gal-mediated sensitization, highlighting the importance of targeted diagnostic testing [7]. Standard skin prick tests often yield negative results in α‑gal patients, whereas in vitro testing for α‑gal-specific IgE provides more reliable detection of sensitization [7]. These findings emphasize AGS as a distinct clinical entity with its hallmark feature of IgE‑mediated delayed anaphylaxis.

Transfusion-Related Implications

AGS has also been increasingly recognized as a contributor to transfusion-related anaphylactic reactions, particularly in the context of exposure to mammalian-derived components present in blood products. Evidence from a retrospective outbreak investigation demonstrated that clustered anaphylactic reactions were associated with group B plasma and platelet transfusions, with affected patients showing elevated α‑gal-specific IgE levels, suggesting that sensitization to α‑gal may trigger immune responses when exposed to these products [15]. These findings expand the clinical relevance of AGS beyond dietary triggers and highlight its role in transfusion medicine. Further supporting this association, a multicenter retrospective analysis identified that ABO‑mismatched plasma and platelet transfusions may increase exposure to α‑gal containing components, providing a potential mechanism for allergic reactions in sensitized individuals [14]. The presence of mammalian‑derived antigens in transfused products may unintentionally activate IgE‑mediated pathways, similar to those seen in delayed food‑related anaphylaxis. This suggests that patients with known or suspected α‑gal sensitization may be at an increased risk for transfusion-related reactions, particularly when exposed to incompatible or higher‑risk plasma components. These studies emphasize the need for screening of AGS in transfusion settings, as well as the implementation of safer matching and transfusion protocols to reduce exposure to materials containing α‑gal. Recognizing this association is critical for preventing unexpected anaphylactic reactions and improving patient safety in clinical practice.

Perioperative Implications

AGS can also create a predisposition to hypersensitivity reactions for patients involving mammalian-derived medical products. Several of the evaluated studies showed associations with heparin products, vaccines, antivenoms, and other biological exposures in patients with AGS. Retrospective analyses demonstrated that although many patients tolerated heparin products, particularly enoxaparin, reactions were more likely among patients with elevated baseline α-gal IgE levels [7]. Similarly, high-dose intravenous heparin administered during cardiac surgery was associated with severe allergic reactions in a subset of patients with significantly elevated α-gal IgE titers [9]. Additional studies identified possible associations between AGS and hypersensitivity reactions following administration of vaccines or biologic therapies containing mammalian-derived components such as gelatin [16]. Comparative studies evaluating mammalian-derived antivenoms also suggested increased concern for hypersensitivity reactions in α-gal endemic regions [19]. These findings emphasize the importance of recognizing hidden mammalian-derived exposures in medical products and considering AGS in patients with unexplained allergic or perioperative reactions.

Management Considerations

Management of AGS primarily relies on long-term dietary modification and avoidance of mammalian‑derived products, as no definitive cure currently exists. An observational study found that patients with AGS must adopt strict dietary restrictions, eliminating foods such as beef, pork, and other mammalian products to prevent IgE‑mediated allergic reactions [17]. These restrictions extend beyond diet, as some medical products and medications may contain hidden α‑gal components. There is an emphasis on the significant impact on the quality of life, as patients must continuously monitor their food intake and potential exposures in both dietary and clinical settings [17,18]. This underscores the importance of long‑term management strategies, including recognition of hidden sources of α‑gal. Overall, treatment of AGS is centered on avoidance and prevention, highlighting the need for continued research into more targeted therapeutic approaches.

Limitations

Several limitations should be considered when interpreting the findings of this review. Sample sizes were often small, particularly in studies evaluating reactions to vaccines, transfusions, antivenoms, and heparin products, which may reduce generalizability. Additionally, diagnostic criteria varied substantially, with some studies relying primarily on α-gal-specific IgE testing while others incorporated clinical history, oral food challenges, or skin testing. This review also excluded non-English studies, animal studies, in vitro studies, review articles, and studies conducted outside the United States, which limits global applicability because the review primarily focused on sensitization associated with *A. americanum* rather than other tick species internationally. Because a formal risk-of-bias assessment was not conducted, as is consistent with scoping review methodology, the relative methodological rigor and quality of evidence among included studies could not be systematically evaluated. However, this review provides a broad characterization of reported AGS manifestations and identifies areas requiring further high-quality research.

Future directions

Improving diagnostic accuracy and standardizing a diagnostic procedure are key directions for the future. Future studies should aim to develop risk-stratification models and evaluate safer alternatives to mammalian‑derived products, thereby improving patient safety during medical procedures. Current treatment relies primarily on strict avoidance of mammalian‑derived foods and products, which can be difficult to maintain and negatively affect daily life. There is a strong need for targeted immunologic treatments, as well as improved food labeling and public health interventions to reduce unintended exposures. Advancing both scientific understanding and clinical management will be critical to improving outcomes for individuals with AGS.

## Conclusions

The findings of this review support that AGS is a unique IgE-mediated hypersensitivity disorder driven by tick-induced sensitization to galactose-α-1,3-galactose rather than a traditional immediate food allergy. Characterized by delayed anaphylaxis occurring several hours following the ingestion of mammalian meat, diagnosis often requires specific IgE testing. Beyond dietary exposures, reported reactions to mammalian-derived pharmaceuticals and blood transfusions, particularly among patients with elevated α-gal IgE levels, highlight the broader clinical implications of AGS. 

Ultimately, understanding AGS as a systemic, tick-induced allergic syndrome rather than a simple food allergy is essential for improving diagnosis, preventing severe reactions, and guiding safer medical management. As AGS continues to emerge as a growing public health concern, these findings emphasize the need for increased clinician awareness, improved screening strategies, and further research into preventive and therapeutic approaches.

## Appendices

**Table 2:** Database queries Table reflecting database-specific search syntax from PubMed, Google Scholar, and Embase.

Database	Date	Search strategy	Result
PubMed	03/22/2026	("galactose-alpha-1,3-galactose"[Title/Abstract] OR "alpha-gal"[Title/Abstract] OR "alpha-gal syndrome"[Title/Abstract]) AND ("Amblyomma americanum"[Title/Abstract] OR "Lone Star tick"[Title/Abstract] OR tick*[Title/Abstract]) AND (sensitization[Title/Abstract] OR anaphylaxis[Title/Abstract] OR "delayed anaphylaxis"[Title/Abstract] OR "red meat allergy"[Title/Abstract] OR heparin[Title/Abstract] OR gelatin[Title/Abstract] OR "bovine-derived"[Title/Abstract] OR "urticaria"[Title/Abstract])	216
Google Scholar	03/22/2026	("alpha-gal syndrome" OR "galactose-alpha-1,3-galactose") AND ("Amblyomma americanum" OR "Lone Star Tick") AND (IgE OR sensitization OR anaphylaxis OR "delayed anaphylaxis") AND (heparin- OR gelatin- OR "bovine-derived" OR urticaria)	488
Embase	03/22/2026	('alpha gal syndrome'/exp OR "alpha-gal syndrome":ti,ab) AND ('amblyomma americanum'/exp OR "Lone Star Tick":ti,ab) AND (IgE:ti,ab OR sensitization:ti,ab) AND (anaphylaxis/exp OR anaphylaxis:ti,ab OR "delayed anaphylaxis":ti,ab OR urticaria:ti,ab)	25
